# Size doesn’t always matter: the case of a voluminous bladder with a diverticulum in an otherwise healthy subject

**DOI:** 10.1007/s40620-022-01359-0

**Published:** 2022-06-07

**Authors:** Vittoria Esposito, Tommaso Ciro Camerota, Marco Colucci, Giuseppe Sileno, Massimo Torreggiani, Ciro Esposito

**Affiliations:** 1 Unit of Nephrology and Dialysis, ICS Maugeri SpA SB, Via Maugeri 10, 27100 Pavia, Italy; 2 Unit of Urology, ICS Maugeri SpA SB, Via Maugeri 10, 27100 Pavia, Italy; 3 Nephrology and Dialysis, Centre Hospitalier Le Mans, Le Mans, France; 4 Department of Internal Medicine and Medical Therapy, University of Pavia, Pavia, Italy

**Keywords:** Overdistended bladder, Bladder diverticulum, Acute kidney injury, Urinary tract infection

**Fig. 1:**
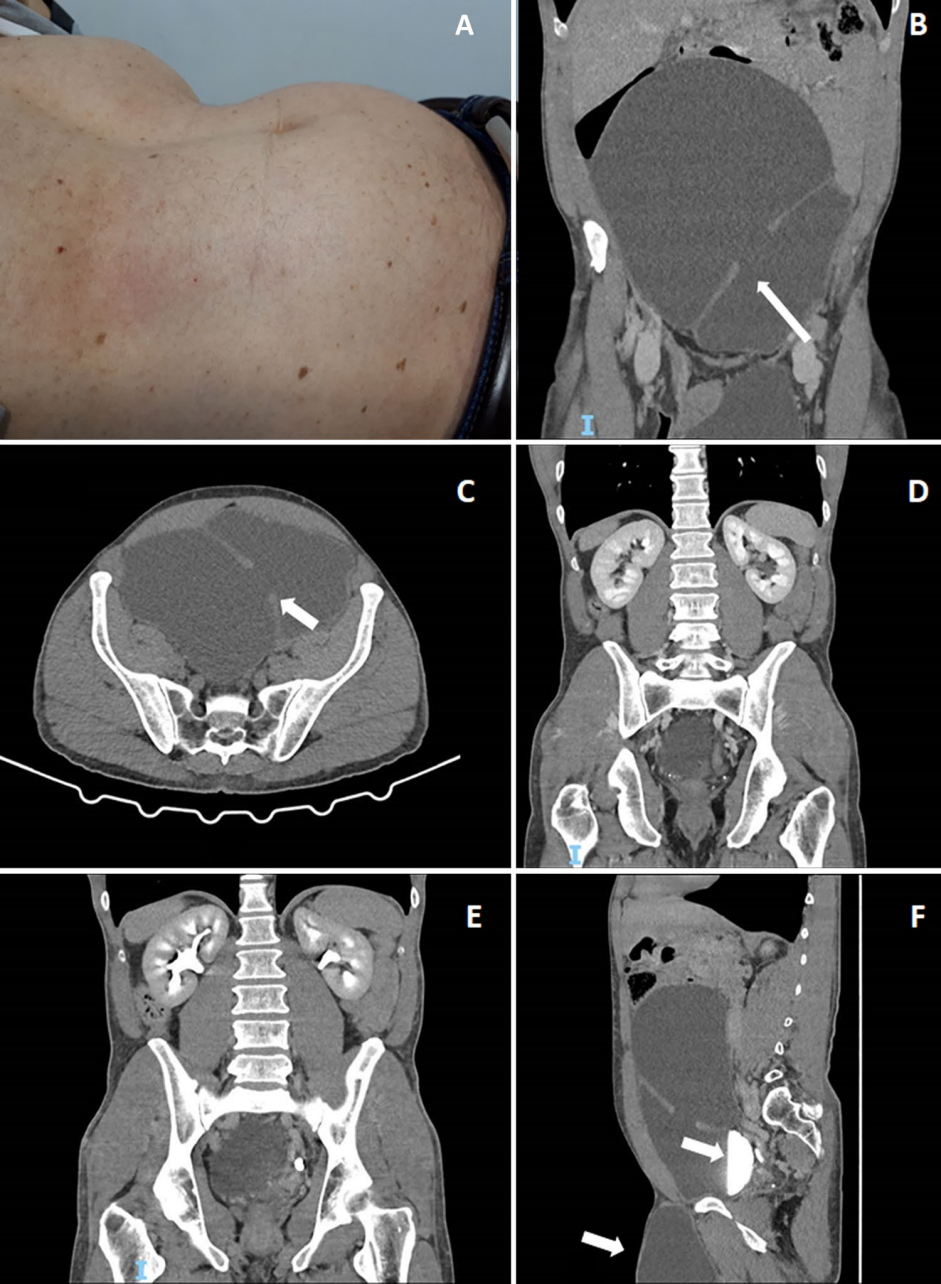
The patient’s bulging abdomen (**A**). Pathological overdistension of the bladder with a large diverticulum (23 cm in diameter); the arrows indicate the origin of the diverticulum from the bladder (**B**, **C**). The diverticulum originates on the bladder right wall reaching the liver cranially with lateral dislocation of the intestinal loops; the arrow indicates the contrast medium in the bladder (**F**). The walls of the bladder appear regular without pathological thickening or endoluminal formations (**C**). Both kidneys show normal size and position, with normal width and structure of the renal parenchyma (**D**). Renal pelvis and calyces are normal and ureters are not dilated (**E**). The cyst in the left scrotal area shows no communication with the bladder (**E** arrow)

A 51-year-old male was referred to a urology outpatient clinic because of a scrotal mass progressively increasing in size. The mass had been detected 10 years before and diagnosed as a spermatic cyst. At that time, surgery was delayed because of its small size. After examining the scrotal mass the urologist’s attention was drawn to the patient’s bulging abdomen (Fig. 1A). An abdominal ultrasound, promptly performed, showed an unusually large bladder. Bladder catheterization led to the excretion of more than 1 L of urine. A CT scan of the abdomen demonstrated pathological overdistension of the bladder with a large diverticulum (23 cm in diameter) (Fig. 1B–C) that originated on the right wall reaching the liver cranially with lateral dislocation of the intestinal loops (Fig. 1F). The walls of the bladder appeared regular without pathological thickening or endoluminal formations (Fig. 1C). Both kidneys were of normal size, structure and position (Fig. 1D). The renal pelvis and calyces were normal and the ureters were not dilated (Fig. 1E). The cyst in the left scrotal area showed no apparent communication with the bladder (Fig. 1E arrow). A retrograde cystourethrography confirmed the overdistended bladder and the absence of communication between the bladder and the spermatic cyst. Renal scintigraphy demonstrated slightly reduced overall renal function (61 ml/min/1.73 m^2^) divided equally between the two kidneys. Lab tests showed serum creatinine 0.85 mg/dl, eGFR 82 ml/min/1.73 m^2^, hemoglobin 13.2 g/dl, total serum proteins 7.0 g/dl, unremarkable urinalysis and absence of proteinuria. Surgery to remove the spermatic cyst and the large bladder diverticulum was scheduled. To our knowledge this is the first report of an overdistended bladder with a huge diverticulum with no renal impairment and absence of hydronephrosis. The giant bladder is commonly observed as a result of urinary tract stenosis [1], neurological disorders [2], or bladder hypotonicity [3] and it is often associated with urinary symptoms such as infections, urinary frequency and abdominal pain, gastrointestinal disturbances and acute kidney injury. In the present patient, bladder overdistension was not related to obstruction or neurological disorders. The patient had never experienced voiding symptoms and his urine stream had always been valid. Thus, our case reminds that increased bladder volume is not always associated with urinary tract obstruction, acute kidney injury, or voiding symptoms especially if bladder pressure is not elevated. Most likely, our patient’s bladder tone was lower than normal and the bladder diverticulum with a “pop-off mechanism” had led to a progressive increase of bladder compliance [4].
